# Correction: A novel biocompatible, simvastatin-loaded, bone-targeting lipid nanocarrier for treating osteoporosis more effectively

**DOI:** 10.1039/d0ra90074e

**Published:** 2020-07-16

**Authors:** Shan Tao, Shao-qing Chen, Wen-tao Zhou, Fang-ying Yu, Lu Bao, Guo-xi Qiu, Qing Qiao, Fu-qiang Hu, Jian-wei Wang, Hong Yuan

**Affiliations:** College of Pharmaceutical Sciences, Zhejiang University 866 Yuhangtang Road Hangzhou 310058 China yuanhong70@zju.edu.cn +86-136-06804049; Anesthesia Department, Zhejiang University School of Medicine, Sir Run Run Shaw Hospital 3 Qingchun East Road Hangzhou 310016 China; Department of Orthopaedics, The Second Affiliated Hospital of Zhejiang University School of Medicine 88 Jiefang Road Hangzhou 310009 China zjuwjw@zju.edu.cn +86-571-87022776 +86-159-58185118

## Abstract

Correction for ‘A novel biocompatible, simvastatin-loaded, bone-targeting lipid nanocarrier for treating osteoporosis more effectively’ by Shan Tao *et al.*, *RSC Adv.*, 2020, **10**, 20445–20459, DOI: 10.1039/D0RA00685H.

The authors regret that incorrect versions of Fig. 7, 9 and 10 were included in the original article. The correct versions of Fig. 7, 9 and 10 are presented below.

**Fig. 7:**
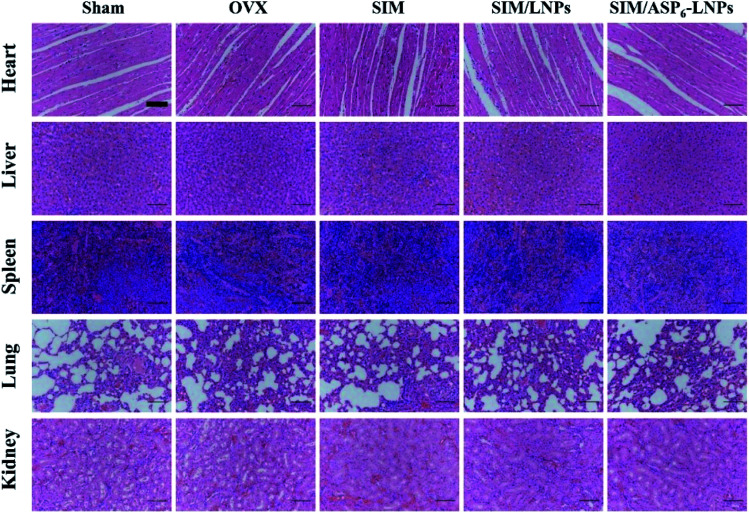
Histological analysis of organs from all experimental groups. H&E staining of heart, liver, spleen, lung, kidney, indicating the carrier has good biocompatibility. Scale bar = 50 μm.

**Fig. 9:**
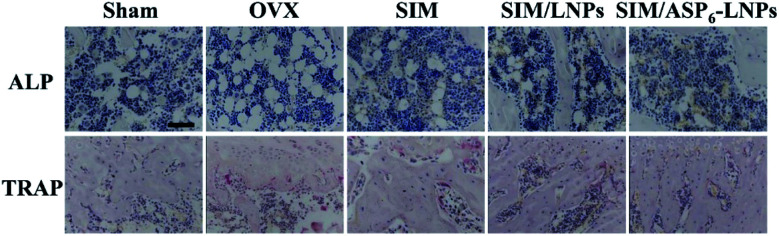
Alkaline phosphatase (ALP) activity (arrows) and tartrate-resistant acid phosphatase (TRAP) assay results (arrowheads) of bone tissue sections. Scale bar = 50 μm. The ALP activity is much more high in SIM/LNPs and SIM/ASP_6_-LNPs groups, while the TRAP activity is the opposite.

**Fig. 10:**
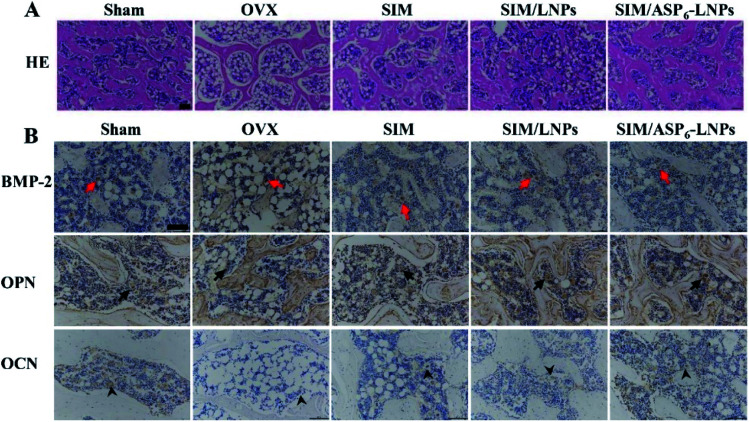
Histological assessment of bone formation in all experimental groups. (A) HE staining of femur bone. Scale bar = 50 μm. Histology of bone in the all experimental groups shows all ovariectomized groups had a higher amount of adipose tissue than Sham group. The trabecular bone is much more prominent in SIM/LNPs and SIM/ASP_6_-LNPs groups. (B) Immunohistochemical staining for BMP-2 in typical newly-formed bone tissue (red arrows) and immunohistochemical staining for the osteogenic markers osteopontin (OPN, arrows) and osteocalcin (OCN, arrowheads). Scale bar = 50 μm. The BMP-2, OPN, OCN are much more prominent in SIM/LNPs and SIM/ASP_6_-LNPs groups.

The Royal Society of Chemistry apologises for these errors and any consequent inconvenience to authors and readers.

## Supplementary Material

